# Approximate Analytical and Numeric Solutions to a Forced Damped Gardner Equation

**DOI:** 10.1155/2022/3240918

**Published:** 2022-05-11

**Authors:** Alvaro H. Salas, S. A. El-Tantawy, Lorenzo J. Martinez H.

**Affiliations:** ^1^Department of Mathematics and Statistics, Universidad Nacional de Colombia, Bogotá, FIZMAKO Research Group, Colombia Sede Manizales; ^2^Department of Physics, Faculty of Science, Port Said University, Port Said 42521, Egypt; ^3^Research Center for Physics (RCP), Department of Physics, Faculty of Science and Arts, Al-Mikhwah, Al-Baha University, Saudi Arabia; ^4^Universidad Nacional de Colombia, Universidad de Caldas, Manizales, Caldas, Colombia

## Abstract

In this paper, some exact traveling wave solutions to the integrable Gardner equation are reported. The ansatz method is devoted for deriving some exact solutions in terms of Jacobi and Weierstrass elliptic functions. The obtained analytic solutions recover the solitary waves, shock waves, and cnoidal waves. Also, the relation between the Jacobi and Weierstrass elliptic functions is obtained. In the second part of this work, we derive some approximate analytic and numeric solutions to the nonintegrable forced damped Gardner equation. For the approximate analytic solutions, the ansatz method is considered. With respect to the numerical solutions, the evolution equation is solved using both the finite different method (FDM) and cubic B-splines method. A comparison between different approximations is reported.

## 1. Introduction

Partial differential equations (PDEs) and ordinary differential equations (ODEs) have received the attention of many researchers of applied mathematics and theoretical physics due to their great role in modeling many natural and engineering phenomena [1–16]. The investigation of the traveling wave solutions (TWSs) to the differential equations plays an important role in the study of nonlinear physical phenomena in different branches of science specially in fluid mechanics, optical fiber, plasma physics, ocean, and sea [1, 2, 7–16]. The following Gardner equation (or combined Korteweg–De Vries (KdV)-modified KdV (mKdV) equation or Extended KdV equation (EKdV)) is one of the most famous equations that was widely used in modeling many physical problems in general and in the physics of plasmas in particular [2, 17–19].

(1) $$\begin{array}{c} {\mathbb{R}}_{1}\equiv \frac{\partial u}{\partial t}+\left(au^{2}+bu\right)\frac{\partial u}{\partial x}+c\frac{\partial^{3}u}{\partial x^{3}}=0, \end{array}$$

where *u* ≡ *u*(*x*, *t*) and (*a*, *b*, *c*) represent the coefficients of the nonlinear and dispersion terms which are function of physical parameters related to the system under study. The Gardner equation (GE) has two nonlinear terms in the quadratic and cubic forms and the dissipative term is of third-order derivative. The GE is an integrable system and Miura transformation connects it to the KdV equation. The GE is a useful model to understand the propagation of acoustic waves in different plasma models [17–19]. This equation can be used for describing the small but finite amplitude of the nonlinear structures that can propagate with phase velocity. However, this equation and some related equations such as KdV equation and modified KdV equation can be used indirectly to describe waves that propagate with the group velocity such as rogue waves and dark and bright solitons. For example, there are many researchers interested in studying waves in plasma physics who used this equation to describe the rouge waves in different plasma models [20–23].

Exact solutions to the GE and some related equations are set up by using various methods [24–45]. Some solutions containing tanh and coth functions are proposed by the extended form of the tanh method [1]. Solitary wave and periodic solutions are constructed by aid of the projective Riccati equations. These solutions have various terms including trigonometric or hyperbolic functions in rational forms. In most published papers, the authors focused on the integrable GE. However, in many physical models there are many effects cannot be ignored such as the collisions between the charged and neutral particles in plasma physics as well as the collisions between the charged particles themselves. Also, some of the external periodic forces can be impacted on the physical model under consideration. If these effects are considered in this case, we can get a nonintegrable evolution equation (forced damped GE). In this paper, we shall proceed to obtain some approximations to the following forced damped GE:

(2) $$\begin{array}{c} {\mathbb{R}}_{2}\equiv \frac{\partial u}{\partial t}+\left(au^{2}+bu\right)\frac{\partial u}{\partial x}+c\frac{\partial^{3}u}{\partial x^{3}}+\gamma \left(t\right)u-F\left(t\right)=0, \end{array}$$

where *γ*(*t*) gives the coefficient of the damping term which arises due to different collisions that take place in plasma physics and *F*(*t*) indicates the external forces. To our knowledge there is no one attempt for studying this equation. Thus, the main goal of this work is to find some approximations for this equation using different analytical and numerical methods.

## 2. Novel Exact Solutions to the Integrable Gardner Equation

In order to obtain some traveling wave solutions (TWSs) to (1), we make the traveling wave transformation *u*=*v*(*ξ*) ≡ *v*, (*ξ*=*kx*+*λt*+*ξ*_0_) which leads to

(3) $$\begin{array}{c} v^{\prime }\left(akv^{2}+bkv+\lambda \right)+ck^{3}v^{\left(3\right)}=0. \end{array}$$

Integrating (3) once over *ξ* and denoting the constant of integration by *d*, we finally get the following Helmholtz–Duffing equation:

(4) $$\begin{array}{c} v^{\prime \prime }+\frac{d}{ck^{3}}+\frac{\lambda }{ck^{3}}v+\frac{b}{2ck^{2}}v^{2}+\frac{a}{3ck^{2}}v^{3}=0. \end{array}$$

Introducing the following ansatz,

(5) $$\begin{array}{c} v\left(\xi \right)=A+B\text{cn}\left(\xi ,m\right), \end{array}$$

into (4) and after direct calculations, we obtain

(6) $$\begin{array}{c} \sum_{j=0}^{3}F_{j}{\text{cn}}^{j}\left(\xi ,m\right)=0, \end{array}$$

where the coefficients *F*_*j*_ are given by

(7) $$\begin{array}{c} F_{0}=\left(2aA^{3}k+3A^{2}bk+6A\lambda +6\;d\right),\\ F_{1}=6B\left(aA^{2}k+Abk+2ck^{3}m-ck^{3}+\lambda \right),\\ F_{2}=3B^{2}k\left(2aA+b\right),\\ F_{3}=2Bk\left(aB^{2}-6ck^{2}m\right). \end{array}$$

Solving the system *S*_*j*_=0 will give us the values of (*A*, *B*, *d*, *λ*) as

(8) $$\begin{array}{c} A=-\frac{b}{2a},\\ B=\pm \sqrt{\frac{6cm}{a}}k,\\ d=\frac{b^{3}k-12abck^{3}\left(2m-1\right)}{24a^{2}},\\ \lambda =\frac{b^{2}k-4ack^{3}\left(2m-1\right)}{4a}. \end{array}$$

Thus, the cnoidal wave solution to GE (1) reads

(9) $$\begin{array}{c} u\left(x,t\right)=-\frac{b}{2a}\pm k\sqrt{\frac{6cm}{a}}\text{cn}\left(kx+\frac{b^{2}k-4ack^{3}\left(2m-1\right)}{4a}t+\xi_{0},m\right). \end{array}$$

For letting *m*⟶1, the soliton solution is recovered.

(10) $$\begin{array}{c} u\left(x,t\right)=-\frac{b}{2a}\pm k\sqrt{\frac{6c}{a}}\sec\;h\left(kx+\frac{b^{2}k-4ack^{3}}{4a}t+\xi_{0}\right). \end{array}$$

It is easy verified that the following solution is a rational solution:

(11) $$\begin{array}{c} u\left(x,t\right)=-\frac{b}{2a}+\frac{k}{1+kx+\left(b^{2}kt/4a\right)}\sqrt{\frac{-6c}{a}}. \end{array}$$

To obtain a traveling wave solution in terms of sn (*ξ*, *m*) and tanh(*ξ*), we make the following substitution:

(12) $$\begin{array}{c} u\left(x,t\right)=A+B\text{sn}\left(\xi ,m\right). \end{array}$$

Inserting the ansatz (12) into GE (1) will give us

(13) $$\begin{array}{c} {\mathbb{R}}_{1}=\frac{1}{B\text{cnd}n}\sum_{j=0}^{2}S_{j}{\text{sn}}^{j}\left(\xi ,m\right)=0, \end{array}$$

where the coefficients *S*_*j*_ (*j*=0,1,2) are defined by

(14) $$\begin{array}{c} S_{0}=aA^{2}k+Abk-ck^{3}-ck^{3}m+\lambda ,\\ S_{1}=\left(2aABk+bBk\right),\\ S_{2}=\left(aB^{2}k+6ck^{3}m\right). \end{array}$$

The solution of the algebraic system *S*_*j*_=0 (*j*=0,1,2) gives the values of coefficients (*A*, *B*, *λ*):

(15) $$\begin{array}{c} A=-\frac{b}{2a},\\ B=\pm k\sqrt{-\frac{6cm}{a}},\\ \lambda =\frac{b^{2}k}{4a}+ck^{3}\left(m+1\right). \end{array}$$

Inserting the values (*A*, *B*, *λ*) given in (15) into ansatz (12), the solution in the form of Jacobi elliptic function sn (*ξ*, *m*) is obtained:

(16) $$\begin{array}{c} u\left(x,t\right)=-\frac{b}{2a}\pm k\sqrt{-\frac{6cm}{a}}\text{sn}\left(kx+\left(\frac{b^{2}k}{4a}+ck^{3}\left(m+1\right)\right)t+\xi_{0},m\right). \end{array}$$

Moreover, for *m*⟶1, the shock wave solution is obtained:

(17) $$\begin{array}{c} u\left(x,t\right)=-\frac{b}{2a}\pm k\sqrt{-\frac{6c}{a}}\tanh\left(kx+\left(\frac{b^{2}k}{4a}+2ck^{3}\right)t+\xi_{0}\right). \end{array}$$

**Remark 1 .**

Equation (4) can be written in the following form:

(18) $$\begin{array}{c} v^{\prime \prime }+r_{0}+r_{1}v+r_{2}v^{2}+r_{3}v^{3}=0. \end{array}$$

The general solution to (18) can be expressed in the terms of Weierstrass elliptic function as following:

(19) $$\begin{array}{c} v\left(\xi \right)=A+\frac{B}{1+C℘\left(\xi +\xi_{0};g_{2},g_{3}\right)}. \end{array}$$

By substituting ansatz solution (19) into the Helmholtz–Duffing equation (18) and after tedious calculations, we get,

(20) $$\begin{array}{c} B=-\frac{6\left(A^{3}r_{3}+A^{2}r_{2}+Ar_{1}+r_{0}\right)}{3A^{2}r_{3}+2Ar_{2}+r_{1}},\\ C=\frac{12}{3A^{2}r_{3}+2Ar_{2}+r_{1}},\\ g_{2}=\left(\begin{array}{c} -3A^{4}r_{3}^{2}-4A^{3}r_{2}r_{3}-6A^{2}r_{1}r_{3}\\ -12Ar_{0}r_{3}+r_{1}^{2}-4r_{0}r_{2} \end{array}\right),\\ g_{3}=\frac{1}{216}\left(\begin{array}{c} 9A^{4}r_{1}r_{3}^{2}-3A^{4}r_{2}^{2}r_{3}-4A^{3}r_{2}^{3}\\ +12A^{3}r_{1}r_{2}r_{3}-6A^{2}r_{1}r_{2}^{2}+\\ 18A^{2}r_{1}^{2}r_{3}-12Ar_{0}r_{2}^{2}+36Ar_{0}r_{1}r_{3}\\ +r_{1}^{3}-6r_{0}r_{1}r_{2}+27r_{0}^{2}r_{3} \end{array}\right). \end{array}$$

The constants *A* and *ξ*_0_ are determined from the initial conditions *v*(0)=*v*_0_ and $v^{\prime }\left(0\right)={\dot{v}}_{0}$. In particular, this gives the general solution to both Duffing and Helmholtz equations, respectively

(21) $$\begin{array}{c} v^{\prime \prime }\left(\xi \right)+r_{1}v\left(\xi \right)+r_{3}v{\left(\xi \right)}^{3}=0,\\ v^{\prime \prime }\left(\xi \right)+r_{1}v\left(\xi \right)+r_{2}v{\left(\xi \right)}^{2}=0. \end{array}$$

We see that the GE (1) admits solutions in terms of the Weierstrass elliptic function. In general, any partial differential equation (pde) can be converted to the ode (18) via the traveling wave transformation, admits TWSs in terms of Weierstrass elliptic function *℘*. Also, Weierstrass function *℘* may be expressed in terms of the Jacobian cn function, which leads to cnoidal wave solutions to GE (1).

The relation between the Jacobian and Weierstrass elliptic functions reads

(22) $$\begin{array}{c} \text{cn}\left(x,m\right)=1-\frac{6}{\left(4m+1\right)\left(\left(12/\left(4m+1\right)\right)℘\left(x;g_{2},g_{3}\right)+1\right)}, \end{array}$$

with

(23) $$\begin{array}{c} g_{2}=\frac{1}{12}\left(16m^{2}-16m+1\right),\\ g_{3}=\frac{1}{216}\left(2m-1\right)\left(32m^{2}-32m-1\right). \end{array}$$

On the other hand,

(24) $$\begin{array}{c} ℘\left(t;g_{2},g_{3}\right)=A+B\frac{1}{1-\text{cn}\left(\sqrt{2}\sqrt[{4}]{\left(3g_{2}/\left(16m^{2}-16m+1\right)\right)t,m}\right)}, \end{array}$$

with

(25) $$\begin{array}{c} A=-\frac{\sqrt{g_{2}}\left(4m+1\right)}{2\sqrt{48m^{2}-48m+3}},\\ B=\sqrt{\frac{3g_{2}}{16m^{2}-16m+1}},\\ m=\frac{1}{4}\left(2-\sqrt{\zeta +3}\right), \end{array}$$

where *ζ* is the least in magnitude root of the following cubic equation:

(26) $$\begin{array}{c} 4\left(g_{2}^{3}-27g_{3}^{2}\right)z^{3}-27g_{2}^{3}z+\left(23g_{2}^{3}+112g_{3}^{2}\right)=0. \end{array}$$

Note that for *g*_2_^3^ − 27*g*_3_^2^=0, we have only one real root,

(27) $$\begin{array}{c} z=\frac{733g_{3}^{2}}{27g_{2}^{3}}. \end{array}$$

Then,

(28) $$\begin{array}{c} m=\frac{1}{2}-\frac{1}{54}\sqrt{730}\approx -3.4282\times 10^{-4}. \end{array}$$

Let *g*_2_^3^ − 27*g*_3_^2^ ≠ 0, the discriminant of the cubic (26) reads

(29) $$\begin{array}{c} \Delta =432\left(g_{2}^{3}-27g_{3}^{2}\right)\left(200g_{2}^{9}+9131g_{3}^{2}g_{2}^{6}+126560g_{3}^{4}g_{2}^{3}+338688g_{3}^{6}\right). \end{array}$$

In this case, our analysis depends on the sign of discriminant (29). Below, we will discuss three cases which depends on the sign of Δ.

### 2.1. First Case: Δ > 0

For Δ > 0, the cubic (26) has three real roots in the following compact form:

(30) $$\begin{array}{c} z_{k}=\frac{3g_{2}^{3/2}}{\sqrt{g_{2}^{3}-27g_{3}^{2}}}\cos\left\{\frac{1}{3}\left[2\pi k+\;\cos^{-1}\left(-\frac{\sqrt{g_{2}^{3}-27g_{3}^{2}}\left(23g_{2}^{3}+112g_{3}^{2}\right)}{27g_{2}^{9/2}}\right)\right]\right\}, \end{array}$$

where *k*=−1,0,1.

### 2.2. Second Case: Δ < 0

For Δ < 0, the cubic (26) has only one real root:

(31) $$\begin{array}{c} \zeta =\frac{{\left(\sqrt{\Delta }-{\left(g_{2}^{3}-27g_{3}^{2}\right)}^{2}\left(23g_{2}^{3}+112g_{3}^{2}\right)\right)}^{2/3}+9g_{2}^{6}-243g_{3}^{2}g_{2}^{3}}{2\left(g_{2}^{3}-27g_{3}^{2}\right)\sqrt[{3}]{\sqrt{\Delta }-{\left(g_{2}^{3}-27g_{3}^{2}\right)}^{2}\left(23g_{2}^{3}+112g_{3}^{2}\right)}}. \end{array}$$

### 2.3. Third Case: Δ=0

Here, suppose that *g*_2_^3^ − 27*g*_3_^2^=0, we have two real roots for cubic (26):

(32) $$\begin{array}{c} z_{1}=\frac{1}{2}\sqrt[{3}]{\frac{23g_{2}^{3}+112g_{3}^{2}}{g_{2}^{3}-27g_{3}^{2}}},\\ z_{2}=-\sqrt[{3}]{\frac{23g_{2}^{3}+112g_{3}^{2}}{g_{2}^{3}-27g_{3}^{2}}}. \end{array}$$

It follows from (24) that the period of Weierstrass elliptic function reads

(33) $$\begin{array}{c} T=2\int_{a}^{+\infty }\frac{\text{d}x}{\sqrt{4x^{3}-g_{2}\;x-g_{3}}}\\ =2\sqrt{2}\sqrt[{4}]{\frac{\zeta }{3g_{2}}K\left(\frac{1}{4}\left(2-\sqrt{\zeta +3}\right)\right)}, \end{array}$$

where *a*  is the greatest real root to the following cubic equation:

(34) $$\begin{array}{c} 4x^{3}-g_{2}x-g_{3}=0. \end{array}$$

Using the identity (24) and the approximation (37), the following approximation is obtained:

(35) $$\begin{array}{c} ℘\left(t;g_{2},g_{3}\right)\approx -\frac{\sqrt{g_{2}}\left(4m+1\right)}{2\sqrt{48m^{2}-48m+3}}+\frac{\sqrt{3g_{2}}}{\sqrt{16m^{2}-16m+1}\left(1-\;\cos_{m}\left(\sqrt{2}\sqrt[{4}]{\left(3g_{2}/\left(16m^{2}-16m+1\right)\right)}t\right)\right)}, \end{array}$$

where *m* is given by (25) and cos_*m*_(*t*):

(36) $$\begin{array}{c} \cos_{m}\left(t\right)=\frac{\sqrt{1+\lambda }\cos\left(\sqrt{1+\lambda }t\right)}{\sqrt{1+\lambda \;\cos^{2}\left(\sqrt{1+\lambda }t\right)}}, \end{array}$$

where $\lambda =\left(1/14\right)\left(\sqrt{m^{2}-144m+144}-m-12\right)$.

The Jacobian elliptic functions cn and sn may be approximated by means of the following expressions:

(37) $$\begin{array}{c} \text{cn}\left(t,m\right)\approx \;\cos_{m}\left(t\right)≔\frac{\sqrt{1+\lambda }\cos\left(\sqrt{1+\lambda }t\right)}{\sqrt{1+\lambda \;\cos^{2}\left(\sqrt{1+\lambda }t\right)}}, \end{array}$$

(38) $$\begin{array}{c} \text{sn}\left(t,m\right)\approx \;\sin_{m}\left(t\right)≔\frac{\sin\left(\sqrt{1+\lambda }t\right)}{\sqrt{1+\lambda \;\cos^{2}\left(\sqrt{1+\lambda }t\right)}}, \end{array}$$

(39) $$\begin{array}{c} \text{dn}\left(t,m\right)\approx {\text{dn}}_{m}\left(t\right)≔\sqrt{1-m\sin_{m}^{2}\left(t\right)}. \end{array}$$

In the following Table 1, we can check the accuracy of the above obtained approximations.

We now give approximate expressions for inverse elliptic cosine and inverse elliptic cosine as follows:

(40) $$\begin{array}{c} {\text{cn}}^{-1}\left(t,m\right)\approx \cos_{m}^{-1}\left(t\right)=\frac{1}{\sqrt{\lambda +1}}\cos^{-1}\left(\frac{t}{\sqrt{\lambda -\lambda t^{2}+1}}\right), \end{array}$$

(41) $$\begin{array}{c} {\text{sn}}^{-1}\left(t,m\right)\approx \sin_{m}^{-1}\left(t\right)=\frac{\mathrm{sgn}\left(t\right)}{\sqrt{\lambda +1}}\cos^{-1}\left(\frac{\sqrt{1-t^{2}}}{\sqrt{1+\lambda t^{2}}}\right), \end{array}$$

where −1 ≤ *t* ≤ 1.

In Table 2, the numerical values of approximations (40) and (41) are displayed.

## 3. Approximate Analytical Solution to the Forced Damped Gardner Equation

Let us suppose that *u*=*v* ≡ *v*(*x*, *t*) is a solution to the GE (1):

(42) $$\begin{array}{c} \frac{\partial v}{\partial t}+\left(av^{2}+bv\right)\frac{\partial v}{\partial x}+c\frac{\partial^{3}\;v}{\partial x^{3}}=0. \end{array}$$

We seek for approximate solution to the forced damped GE (2) in the ansatz form:

(43) $$\begin{array}{c} u=f\left(t\right)v\left(xg\left(t\right),h\left(t\right)\right)+\varphi \left(t\right). \end{array}$$

The functions *f*, *g*, *h*, and *φ* are to be determined later. Plugging ansatz (43) into (2) and taking the following value of *∂v*/*∂t* into account:

(44) $$\begin{array}{c} \frac{\partial v}{\partial t}=-av^{2}\frac{\partial v}{\partial x}-bv\frac{\partial v}{\partial x}-c\frac{\partial^{3}v}{\partial x^{3}}, \end{array}$$

we finally obtain

(45) $$\begin{array}{c} {\mathbb{R}}_{2}=\left(\varphi^{\prime }\left(t\right)+\gamma \left(t\right)\varphi \left(t\right)-F\left(t\right)\right)\\ +f\left(t\right)v_{x}\left(g\left(t\right)\varphi \left(t\right)\left(b+a\varphi \left(t\right)\right)+xg^{\prime }\left(t\right)\right)\\ +av^{2}f\left(t\right)\left(f{\left(t\right)}^{2}g\left(t\right)-h^{\prime }\left(t\right)\right)v_{x}\\ +cf\left(t\right)\left(g{\left(t\right)}^{3}-h^{\prime }\left(t\right)\right)v_{xxx}\\ +\left(f\left(t\right)\gamma \left(t\right)+f^{\prime }\left(t\right)\right)v\\ +\left(f\left(t\right)g\left(t\right)\left(b+2a\varphi \left(t\right)\right)-bh^{\prime }\left(t\right)\right)vf\left(t\right)v_{x}. \end{array}$$

The last expression suggests the choices:

(46) $$\begin{array}{c} \varphi^{\prime }+\gamma \left(t\right)\varphi -F=0,\\ f^{2}g-h^{\prime }=0,\\ f\gamma \left(t\right)+f^{\prime }=0,\\ g=f. \end{array}$$

We will choose the functions *f*, *g*, *h*, and *φ* so that

(47) $$\begin{array}{c} f\left(0\right)=g\left(0\right)=1,\\ h\left(0\right)=\varphi \left(0\right)=0. \end{array}$$

Solving system (46) and (47), we obtain the following solutions:

(48) $$\begin{array}{c} f\left(t\right)=g\left(t\right)=\exp\left(-\int_{0}^{t}\gamma \left(\tau \right)\text{d}\tau \right). \end{array}$$

(49) $$\begin{array}{c} h\left(t\right)=\int_{0}^{t}\exp\left(-3\int_{0}^{\eta }\gamma \left(\tau \right)\text{d}\tau \right)\text{d}\eta . \end{array}$$

(50) $$\begin{array}{c} \varphi \left(t\right)=\exp\left(\int_{0}^{t}-\gamma \left(\tau \right)\text{d}\tau \right)\int_{0}^{t}\exp\left(-\int_{0}^{\eta }-\gamma \left(\tau \right)\text{d}\tau \right)F\left(\eta \right)\text{d}\eta . \end{array}$$

Thus, an approximate analytical solution to the damped and forced GE will be

(51) $$\begin{array}{c} u\left(x,t\right)=\exp\left(-\int_{0}^{t}\gamma \left(\tau \right)\text{d}\tau \right)V\left(x\;\exp\left(-\int_{0}^{t}\gamma \left(\tau \right)\text{d}\tau \right),\int_{0}^{t}\exp\left(-3\int_{0}^{\eta }\gamma \left(\tau \right)\text{d}\tau \right)\text{d}\eta \right)+\varphi \left(t\right), \end{array}$$

where *φ*=*φ*(*t*) can found from (50) and *V*(*x*, *t*)=*v*(*x*, *t*) is any analytic solution to the integrable GE (42).

## 4. FDM for Analyzing the Forced Damped Gardner Equation

To apply FDM for analyzing (2), we first write this equation in the following initial value problem (i.v.p.):

(52) $$\begin{array}{c} \left\{\begin{array}{c} {\mathbb{R}}_{2}=0,\\ u\left(x,0\right)=f\left(x\right), \end{array}\right. \end{array}$$

where *X*_*i*_ ≤ *x* ≤ *X*_*f*_ and 0 ≤ *t* ≤ *T*_*f*_.

The space-time domain are divided into subintervals with uniform size as

(53) $$\begin{array}{c} x_{i}=X_{i}+i\Delta x,\;\Delta x=\frac{X_{f}-X_{i}}{m},\\ t_{j}=j\Delta t,\;\Delta t=\frac{T_{f}}{n}, \end{array}$$

where *m* and *n* are integer numbers.

According to the FDM, the following derivatives formulas are introduced:

(54) $$\begin{array}{c} \frac{\partial u}{\partial t}\left(x_{i},t_{j}\right)=\frac{u_{i,j-2}-8u_{i,j-1}+8u_{i,j+1}-u_{i,j+2}}{12\Delta t},\\ \frac{\partial u}{\partial x}\left(x_{i},t_{j}\right)=\frac{u_{i-2,j}-8u_{i-1,j}+8u_{i+1,j}-u_{i+2,j}}{12\Delta x},\\ \frac{\partial^{3}u}{\partial x^{3}}\left(x_{i},t_{j}\right)=\frac{u_{i-3,j}-8u_{i-2,j}+13u_{i-1,j}-13u_{i+1,j}+8u_{i+2,j}-u_{i+3,j}}{8\Delta x^{3}}. \end{array}$$

In the case, when *i* < 0 or *i* > *m* or *j* < 0 or *j* > *n*, we define $u_{i,j}=u\left(x_{i},t_{j}\right)=\tilde{u}\left(x_{i},t_{j}\right)$, where $\tilde{u}=\tilde{u}\left(x,t\right)$ is the analytical approximation defined in Section 3.

Now, we solve the following system of the nonlinear algebraic equations:

(55) $$\begin{array}{c} \frac{u_{i,j-2}-8u_{i,j-1}+8u_{i,j+1}-u_{i,j+2}}{12\Delta t}+\left(au_{i,j}^{2}+bu_{i,j}\right)\frac{u_{i-2,j}-8u_{i-1,j}+8u_{i+1,j}-u_{i+2,j}}{12\Delta x}\\ +c\frac{u_{i-3,j}-8u_{i-2,j}+13u_{i-1,j}-13u_{i+1,j}+8u_{i+2,j}-u_{i+3,j}}{8\Delta x^{3}}+\gamma \left(t_{j}\right)u_{i,j}=F\left(t_{j}\right), \end{array}$$

with *u*_*i*,0_=*f*(*x*_*i*_) and *i*=0,1,2,…, *m* and *j*=0,1,2,…, *n*.

If we already solved system (55), then, we may construct an interpolation function with the data (*x*_*i*_, *t*_*j*_, *u*_*i*,*j*_) for *i*=0,1,2,…, *m* and *j*=0,1,2,…, *n*. This interpolation function will represent the approximate numerical solution to the evolution equation. Another numerical solution may be obtained using the NDSolve Mathematica command.

## 5. Cubic Splines (Odd-order B-splines) for Analyzing the Forced Damped Gardner Equation

The general odd B-splines of (2*r* − 1)− order are defined as

(56) $$\begin{array}{c} \varphi_{i}=\\ \frac{1}{h^{\left(2r-1\right)}}\left[\begin{array}{c} \sum_{j=0}^{r-1}\left(\sum_{k=0}^{j}{\left(-1\right)}^{k}\left(\begin{array}{c} 2r\\ k \end{array}\right){\left(x-\xi_{i-\left(r-k\right)}\right)}^{2r-1}\right)\chi_{i-\left(r-j\right)}\\ +\sum_{j=0}^{r-1}\left(\sum_{k=0}^{j}{\left(-1\right)}^{k}\left(\begin{array}{c} 2r\\ k \end{array}\right){\left(\xi_{i+r-k}-x\right)}^{2r-1}\right)\chi_{i+\left(r-j\right)-1} \end{array}\right], \end{array}$$

with *h*=(*b* − *a*)/*n*, *ξ*_*i*_=*a*+*ih*, and *χ*_*s*_ ≡ *χ*_*s*_(*x*)=*χ*_[*a*+*sh*, *a*+(*s*+1)*h*)_. Here, *χ*_*s*_(*x*)=1 for *a*+*sh* ≤ *x* < *a*+(*s*+1)*h* and 0 otherwise and *φ*_*i*_ ≡ *φ*_*i*_(*x*).

Note that for *r*=2, we obtain the so-called cubic B-splines as follows:

(57) $$\begin{array}{c} \varphi_{i}=\frac{1}{h^{3}}\left(\begin{array}{c} Z_{0}\chi_{i-2}\left(x\right)+Z_{1}\chi_{i-1}\left(x\right)\\ +Z_{2}\chi_{i}\left(x\right)+Z_{3}\chi_{i+1}\left(x\right) \end{array}\right), \end{array}$$

with

(58) $$\begin{array}{c} Z_{0}={\left(x-\xi_{i-2}\right)}^{3},\\ Z_{1}=\left({\left(x-\xi_{i-2}\right)}^{3}-4{\left(x-\xi_{i-1}\right)}^{3}\right),\\ Z_{2}=\left({\left(\xi_{i+2}-x\right)}^{3}-4{\left(\xi_{i+1}-x\right)}^{3}\right),\\ Z_{3}={\left(\xi_{i+2}-x\right)}^{3}, \end{array}$$

Assuming that *u*(*x*, *t*)=∑_*k*=*i*−1_^*n*+1^*δ*_*k*_(*t*)*φ*_*k*_(*x*) and with the help of Table 3, we get,

(59) $$\begin{array}{c} u_{t}\left(\xi_{i},t\right)=h^{-6}\left(\delta_{i-1}^{\prime }\left(t\right)+4\delta_{i}^{\prime }\left(t\right)+\delta_{i+1}^{\prime }\left(t\right)\right),\\ u\left(\xi_{i},t\right)=h^{-6}\left(\delta_{i-1}\left(t\right)+4\delta_{i}\left(t\right)+\delta_{i+1}\left(t\right)\right),\\ u_{x}\left(\xi_{i},t\right)=3h^{-5}\left(\delta_{i+1}\left(t\right)-\delta_{i-1}\left(t\right)\right),\\ u_{xx}\left(\xi_{i},t\right)=6h^{-4}\left(\delta_{i-1}\left(t\right)-2\delta_{i}\left(t\right)+\delta_{i+1}\left(t\right)\right),\\ u_{xxx}\left(\xi_{i},t\right)=6h^{-3}\left(-\delta_{i-1}\left(t\right)+3\delta_{i}\left(t\right)-3\delta_{i+1}\left(t\right)+\delta_{i+2}\left(t\right)\right). \end{array}$$

These formulas may be employed for solving PDEs like KdV, KdV-Burgers, MKdV, Gardner, and many third-order PDEs arising in different branches of science specially plasma physics.

Now, in order to solve the i.v.p. (52), we must solve the following system of nonlinear odes:

(60) $$\begin{array}{c} \delta_{i-1}^{\prime }\left(t\right)+4\delta_{i}^{\prime }\left(t\right)+\delta_{i+1}^{\prime }\left(t\right)-\frac{6\gamma \left(\delta_{i-1}\left(t\right)-3\delta_{i}\left(t\right)+3\delta_{i+1}\left(t\right)-\delta_{i+2}\left(t\right)\right)}{h^{3}}-\\ \frac{3}{h}\left[\begin{array}{c} \left(\delta_{i-1}\left(t\right)-\delta_{i+1}\left(t\right)\right)\left(\delta_{i-1}\left(t\right)+4\delta_{i}\left(t\right)+\delta_{i+1}\left(t\right)\right)\\ \left(\alpha +\beta \left(\delta_{i-1}\left(t\right)+4\delta_{i}\left(t\right)+\delta_{i+1}\left(t\right)\right)\right) \end{array}\right]=F_{0}\cos\left(\omega t\right), \end{array}$$

where *i*=−1,0,…, *n*, *n*+1, *δ*_*j*_(*t*)=0 for *j* < −1 or *j* > *n*+1. We must choose the value of *h*=(*b* − *a*)/*n* in order to get the least residual error as possible.

## 6. Analysis and Discussion

We have obtained some analytical and numerical approximations the integrable GE (1) and nonintegrable forced damped GE (2). For analyzing the obtained solutions, we start by an exact solution to integrable GE (1). Some exact solutions to GE (1) such as the cnoidal wave solution given in (8) and the soliton solution given in (9) are introduced during the analysis the approximate solutions to the nonintegrable forced damped GE (2). Also, the following exact soliton solution is introduced to analyze (2).

(61) $$\begin{array}{c} u=\frac{6ck^{2}}{b+\sqrt{b^{2}+6ack^{2}}\cosh\left(kx-ck^{3}t\right)}. \end{array}$$

**Figure 1:**
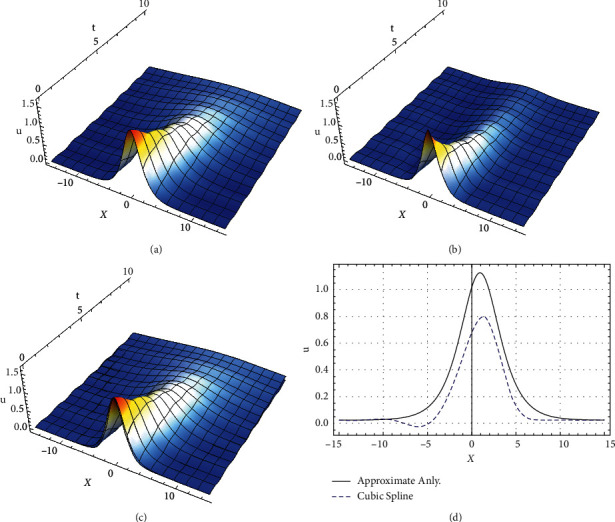
Profile of the approximate analytic soliton solution and the cubic B-splines soliton solution to the forced damped Gardner equation (2) is presented.

Now, based on soliton solution (61) and according to the values (*a*, *b*, *c*, *γ*(*t*), *F*_0_, *ω*, *k*)=(1,1,1,0.2, 0.1, 4,1), the profile of the approximate analytic soliton solution and the cubic B-splines soliton solution to the forced damped GE (2) is, respectively, presented in Figure 1. Also, the global residual errors in the whole domain for both approximate analytic solution and the numerical approximation using cubic B-splines for *n*=20 are, respectively, estimated as *L*_*r*_=0.630565 and *L*_*r*_=0.757672. Note that the accuracy of the approximations depends on the values of physical parameters and the chosen exact solution.

## 7. Conclusion

In this work, some novel exact solutions to the integrable Gardner equation (GE) and approximations to the nonintegrable forced damped Gardner equations have been obtained. The most important obtained results can be briefly summarized in the following points:

1. In the first part, the integrable GE was reduced to the Helmholtz–Duffing equation using traveling wave transformation. After that, some exact solutions have been derived using the ansatz method. The obtained solutions have been derived in the form of Jacobi and Weierstrass elliptic functions. Moreover, the relation between Jacobi and Weierstrass elliptic functions has been presented. The obtained solutions can be recovered cnoidal waves, solitary waves, and shock waves to the GE.
2. In the second part, general formula for the approximate analytical solution to the forced damped GE has been derived in detail. This solution can be recovered from many nonlinear solutions that can be created and propagated in plasma physics. Based on this formula, the characteristics of many nonlinear structures in plasma physics such as solitary waves, shock waves, and cnoidal waves can be investigated.
3. In the third part, the evolution equation (the forced damped GE) has been analyzed using FDM in order to obtain an approximate numerical solution.
4. In the fourth-part, the cubic splines (Odd-order B-splines) were employed for analyzing the forced damped GE numerically.

Finally, the obtained solutions can help all researchers who are interested by studying the nonlinear structures in fluid mechanics, optical fiber, physics of plasmas, ocean and seas, and water tank waves.

## Figures and Tables

**Table 1:** Numerical values of approximations (37) and (38).

*m*	max_−*T*≤*t*≤*T*_|cn(*t*, *m*) − cos_*m*_(*t*)|	max_−*T*≤*t*≤*T*_|sn(*t*, *m*) − sin_*m*_(*t*)|
−1	0.00663668	0.00397374
−0.9	0.00561563	0.00337397
−0.8	0.00463848	0.00280023
−0.7	0.00371582	0.0022567
−0.6	0.00285937	0.00174918
−0.5	0.0020823	0.00128479
−0.4	0.00139952	0.000872175
−0.3	0.000828108	0.00052203
−0.2	0.000387927	0.000247775
−0.1	0.000102461	0.0000664333
0	0	0
0.1	0.000115401	0.0000775637
0.2	0.00049276	0.000338399
0.3	0.00118967	0.000837207
0.4	0.00228433	0.0016531
0.5	0.00388836	0.00290054
0.6	0.0061719	0.00474848
0.7	0.00941955	0.00748209
0.8	0.0141814	0.0116506
0.9	0.0218477	0.018634

**Table 2:** Numerical values of approximations (40) and (41).

*m*	max_−1≤*t*≤1_|cn^−1^(*t*, *m*) − cos_*m*_^−1^(*t*)|	max_−1≤*t*≤1_|sn^−1^(*t*, *m*) − sin_*m*_^−1^(*t*)|
0.	0	0
0.1	0.000186339	0.000156673
0.2	0.000858447	0.000722853
0.3	0.00225528	0.00190314
0.4	0.00476205	0.00403892
0.5	0.0090332	0.00771515
0.6	0.0162603	0.0140741
0.7	0.0288284	0.0255169
0.8	0.0521858	0.0482857
0.9	0.105981	0.105797

**Table 3:** Formulas for cubic splines at nodes.

*∗*	*φ*_*i*_	*φ*_*i*_′	*φ*_*i*_^″^	*φ*_*i*_^(3)^
*ξ*_*i*−2_	0	0	0	6/*h*^3^
*ξ*_*i*−1_	1	3/*h*	6/*h*^2^	−(18/*h*^3^)
*ξ*_*i*_	4	0	−(12/*h*^2^)	18/*h*^3^
*ξ*_*i*+1_	1	−(3/*h*)	6/*h*^2^	−(6/*h*^3^)

## Data Availability

No data were used to support this paper.

## Appendix

### A. Appendix I. The coefficients *W*_*j*_ (*j*=0,1,…, 8)

(A.1)
$$\begin{array}{c} W_{0}=-6gf_{x}g_{x}\left(aA^{2}f^{2}g_{x}+Abf^{3}+Abfg^{2}-18cf^{2}g_{x}+6cg^{2}g_{x}\right),\\ W_{1}=3f_{x}^{2}\left(2aA^{2}fg^{2}g_{x}+Abf^{2}g^{2}+Abg^{4}+12cf^{3}g_{x}-36cfg^{2}g_{x}\right),\\ W_{2}=-2gf_{x}^{3}\left(aA^{2}g^{2}+18cf^{2}-6cg^{2}\right),\\ W_{3}=+2fg_{x}^{3}\left(aA^{2}f^{2}-6cf^{2}+18cg^{2}\right),\\ W_{4}=3Abf^{2}\left(f^{2}+g^{2}\right)g_{x}^{2},\\ W_{5}=-18c\left(f^{2}+g^{2}\right)f_{xx}\left(f^{2}g_{x}-2fgf_{x}-g^{2}g_{x}\right),\\ W_{6}=-18c\left(f^{2}+g^{2}\right)g_{xx}\left(f^{2}f_{x}-g^{2}f_{x}+2fgg_{x}\right),\\ W_{7}=-6cg{\left(f^{2}+g^{2}\right)}^{2}f_{xxx}+6cf{\left(f^{2}+g^{2}\right)}^{2}g_{xxx},\\ W_{8}=-6g{\left(f^{2}+g^{2}\right)}^{2}f_{t}+6f{\left(f^{2}+g^{2}\right)}^{2}g_{t}. \end{array}$$

### B. Appendix II: The coefficients *Y*_*j*_ (*j*=0,1,2)

(B.1)
$$\begin{array}{c} Y_{0}=2\left(aA^{2}-24c\right)D_{x}{\left(f\cdot g\right)}^{3}-3Ab\left(f^{2}+g^{2}\right)D_{x}{\left(f\cdot g\right)}^{2},\\ Y_{1}=-18c\left(f^{2}+g^{2}\right)D_{x}\left(f\cdot g\right)\left(D_{xx}\left(f\cdot f\right)+D_{xx}\left(g\cdot g\right)\right),\\ Y_{2}=6c{\left(f^{2}+g^{2}\right)}^{2}D_{xxx}\left(f\cdot g\right)+6{\left(f^{2}+g^{2}\right)}^{2}D_{t}\left(f\cdot g\right). \end{array}$$
